# Regression adjusted colocalisation colour mapping (RACC): A novel biological visual analysis method for qualitative colocalisation analysis of 3D fluorescence micrographs

**DOI:** 10.1371/journal.pone.0225141

**Published:** 2019-11-11

**Authors:** Rensu P. Theart, Ben Loos, Thomas R. Niesler

**Affiliations:** 1 Department of Electrical and Electronic Engineering, Stellenbosch University, Stellenbosch, Western Cape, South Africa; 2 Department of Physiological Sciences, Stellenbosch University, Stellenbosch, Western Cape, South Africa; University of Houston, UNITED STATES

## Abstract

The qualitative analysis of colocalisation in fluorescence microscopy is of critical importance to the understanding of biological processes and cellular function. However, the degree of accuracy achieved may differ substantially when executing different yet commonly utilized colocalisation analyses. We propose a novel biological visual analysis method that determines the correlation within the fluorescence intensities and subsequently uses this correlation to assign a colourmap value to each voxel in a three-dimensional sample while also highlighting volumes with greater combined fluorescence intensity. This addresses the ambiguity and variability which can be introduced into the visualisation of the spatial distribution of correlation between two fluorescence channels when the colocalisation between these channels is not considered. Most currently employed and generally accepted methods of visualising colocalisation using a colourmap can be negatively affected by this ambiguity, for example by incorrectly indicating non-colocalised voxels as positively correlated. In this paper we evaluate the proposed method by applying it to both synthetic data and biological fluorescence micrographs and demonstrate how it can enhance the visualisation in a robust way by visualising only truly colocalised regions using a colourmap to indicate the qualitative measure of the correlation between the fluorescence intensities. This approach may substantially support fluorescence microscopy applications in which precise colocalisation analysis is of particular relevance.

## Introduction

Fluorescence microscopy is a major driving force in modern biology and medicine, offering steadily increasing resolution and power of analysis. In such analyses, colocalisation, the geometric codistribution of two fluorescence colour channels (often referred to as signals), provides critical information indicating whether two proteins or structures of interest associate with one another. This is important for the understanding of biological processes and cellular functions. However, the objective is usually not merely to consider the spatial overlap of two colour channels, since this would include coincidental overlap. Instead, it is of much greater importance to consider the correlation, or the proportional overlap, of two colour channels within and between structures [1]. Therefore, for many colocalisation applications, it is desirable to accurately quantify the degree of colocalisation in the sample as well as to assess the location and intensity thereof clearly.

A common approach to quantifying colocalisation is the calculation of several colocalisation metrics, each of which highlights a particular aspect of the colocalisation and signal distribution throughout the sample or within an isolated region of interest (ROI). Some of the most notable and widely utilized among these metrics are the Pearson correlation coefficient (PCC), the Manders Overlap coefficient (MOC) and the Manders correlation coefficient (MCC) [2]. These metrics calculate a single value that provides an indication of the overall correlation between the underlying colocalised fluorescence intensities over the analysis region as a whole. Although these measures are effective for the comparison of colocalisation between samples, especially when coupled with ROI selection, they are less suitable to convey any spatial information. Therefore, since sample investigations often require an understanding of how a fluorescence signal distributes throughout intracellular regions, another frequent approach to the analysis of colocalisation is by means of visualisation. Often this is achieved by overlaying the two fluorescence channel images and observing regions of overlap. For example, in the case of a red and green channel combination, the overlapping regions will be visualised in yellow. Although this approach provides a rapid overview of potentially colocalised signals, the ability to observe such yellow areas is highly dependent on the relative signal intensity of each channel. This is problematic since the intensity dynamics are rarely similar across different samples acquired through fluorescence microscopy.

Another common approach in the life sciences is to show the overlay of the fluorescence intensities together with a binary mask of the colocalised signal distribution. This binary mask is either shown by itself or superimposed on the fluorescence intensities as a single colour (often white) [3]. In this visualisation approach only the location of the colocalisation is shown. Limited or no indication is provided of the underlying intensities resulting in the observed colocalisation, or of the extent of the correlation between the channels. Lastly, visualisation of spatial colocalisation is most often performed two-dimensionally (2D) and only limited work has been undertaken to allow visualisation in three-dimensional (3D) space [4–6].

In this paper, we aim to address the above challenges, especially the limitations associated with showing the colocalised voxels only as a binary mask, by using a new approach that models the correlation of the underlying colocalised fluorescence signals spatially and visualises its distribution in three dimensions. With this newly proposed biological visual analysis method, which we refer to as *regression adjusted colocalisation colour mapping* (RACC), we aim to improve the 3D spatial interpretation of the colocalisation signal distribution within a sample in a robust way by capturing both the underlying channel intensities as well as their correlation. We demonstrate how this enhances the visualisation of colocalisation by analysing both synthetic data as well as biological samples, acquired through confocal as well as super-resolution techniques, with a focus on vesicle as well as tubulin network interactions. We further highlight the analytical strengths of RACC by integrating it with a recently developed virtual reality enabled 3D ROI selection tool [7]. RACC is intended as a complementary approach to existing colocalisation metrics. It has the specific aim of contributing an understanding of how the correlation between the underlying fluorescence intensities varies spatially. Our implementation of RACC is available for download.

### Existing colocalisation intensity models

Several existing approaches consider the spatial quantification of the colocalisation in a sample, with each designed to highlight a certain aspect of the colocalisation. In order to contextualize the derivation of RACC, these models will be discussed briefly.

With the aim of improving the identification of colocalised structures at a subcellular level, enhanced colocalisation visualisation was pursued using a specifically designed dual-channel look-up table (LUT) that maps fluorescence channel visualisations from Texas Red to magenta and FITC to cyan [8]. Red was used to indicate colocalised voxels that have a greater intensity in the Texas Red channel, green was used to indicate colocalised voxels that have greater intensities in the FITC channel and yellow was used when both intensities were similar. In this way the relative intensities of the fluorescence channels in the colocalised voxel could be better discriminated [3].

Building on this work, two subsequent colocalisation visualisation methods were proposed, each having the advantage of not relying on the balanced staining of cells to ensure similar fluorescent signal intensities in both channels [9]. These methods were named the covariance method, which is a spatial representation of the PCC, and the multiply method, which is a spatial representation of MOC. Pixels having the greatest influence on the metrics are identified by visualising only those falling within the 99th percentile. In a similar way, several colocalisation metrics as well as the product of the PCC and MOC spatial maps can be visualised in what is referred to as a mixed map [10]. This mixed map is then used in an iterative classification process to generate a map that is a better approximation of the true colocalisation, as opposed to the coincidental colocalisation, thereby reducing the visualisation of false positives.

A currently very prominent approach to the visualisation of colocalisation provides a spatial representation of the correlation between two fluorescent signals [11], similar to the spatial representation of the PCC described above. For each pixel in the sample, a quantity termed the *normalized mean deviation product* (nMDP) is calculated. The nMDP is calculated for each pixel in the image and is defined as:

$${\text{nMDP}}_{p_{i}}=\frac{\left(x_{i}-\bar{x}\right)\left(y_{i}-\bar{y}\right)}{\left(x_{p_{\text{max}}}-\bar{x}\right)\left(y_{p_{\text{max}}}-\bar{y}\right)}$$ (1)

with *x*_*i*_ and *y*_*i*_ representing the fluorescence intensities of the two colour channels for the *i*^th^ pixel in the sample image, $\bar{x}$ and $\bar{y}$ representing the mean channel intensities and *x*_max_ and *y*_max_ representing the maximum channel intensities.

To calculate the mean and maximum channel intensities required in Eq 1, a Sobel filter is applied to each colour channel of the image in order to make a region of interest (ROI) selection. In this way background intensities are removed independently for each colour channel of the image. The use of a Sobel filter for background subtraction is however not required for nMDP and the authors note that other methods, such as manual intensity thresholding, are equally applicable. In the numerator of Eq 1, the product of the deviations from the mean within the ROI is calculated for fluorescence channels *x* and *y*. This product is then normalized by the product of the deviations of the maximum intensities from the respective means over the entire image. Using this equation, a new pseudo-colour image is generated that represents the degree to which colocalisation or non-colocalisation occurs at each pixel. The nMDP value can be either positive or negative, based on the intensity of the pixel relative to the mean. In this way, positive values indicate that the pixel is colocalised and negative values indicate that it is not.

All the above methods aim to improve the spatial analysis of colocalisation by augmenting the standard visualisation of the overlap between two fluorescence channels in their original colours. Some of these attempt to introduce a visual representation of the correlation between the channels into the sample visualisation, by assigning a colour to each voxel based on its individual contribution to the PCC. Since the colocalisation metrics were not designed to be visualised directly, this can however introduce inconsistencies into the interpretation of the visualisation, as we will demonstrate. We also demonstrate how these inconsistencies are avoided by RACC. We will employ RACC to explore synthetic data as well as biological samples acquired through super resolution structured illumination microscopy (SR-SIM) and demonstrate the advantages of analysing colocalisation in this manner. Since nMDP is most closely related to RACC in design and purpose, we will use this technique as a reference throughout.

## Regression adjusted colocalisation colour mapping (RACC)

When investigating colocalisation, usually the correlation and not merely the co-occurrence of fluorescence channel intensities is of interest. The intensity of a fluorescence channel of an image is dependent mainly on the abundance of the fluorochrome in that region of the cell. Therefore, when two proteins or structures of interest associate with one another, their fluorescent signals will usually have similar intensities in the same voxels of the image. One of the main aims of a colocalisation model is to spatially visualise the correlation between the two fluorescence channel intensities, specifically the degree to which colocalised voxels are *positively* correlated. The two fluorescence intensities, for each voxel, can be represented by two random variables *X* and *Y*. In current micrographs, *X* and *Y* are usually discrete, with integer values between 0 and 255. For a given voxel *i* in the sample, this pair of intensities will henceforth be referred to as the *colocalisation intensity* **q**_*i*_ and defined as:

$$q_{i}=\left(x_{i},y_{i}\right)$$ (2)

For a spatial visualisation of the correlation between fluorescence channels, the colocalisation intensity **q**_*i*_ of each voxel should be assigned a colourmap value. This corresponds to a mapping from a 2D to a 1D subspace. Most existing methods, including nMDP (Eq 1), achieve this by a direct spatial visualisation of the PCC.

### Direct visualisation of the PCC

The PCC can be estimated for *X* and *Y*, over *N* intensity bearing voxels in the ROI, using the sample correlation coefficient, as follows:

$$PCC={\hat{\rho }}_{X,Y}=\frac{\sum_{i=1}^{N}\left(x_{i}-\bar{x}\right)\times \left(y_{i}-\bar{y}\right)}{\sqrt{\sum_{i=1}^{N}{\left(x_{i}-\bar{x}\right)}^{2}\times \sum_{i=1}^{N}{\left(y_{i}-\bar{y}\right)}^{2}}}$$ (3)

In Eq 3, the denominator functions as a normalization factor. The individual terms of the summation in the numerator can be viewed as an indication of how much the colocalisation intensity **q**_*i*_ of each voxel in the ROI contributes to the estimated correlation coefficient ${\hat{\rho }}_{X,Y}$. Hence some previous work visualised the normalized contribution of each voxel to ${\hat{\rho }}_{X,Y}$ directly using a colourmap [9–11]. This approach has some disadvantages, however.

**Fig 1:**
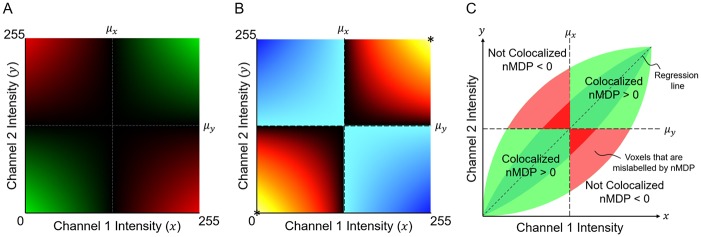
Direct visualisation of the PCC. The relative contribution of each voxel’s colocalisation intensity **q**_*i*_ to the correlation coefficient is determined by the individual terms of the summation in the numerator of Eq 3. With *x* and *y* axes representing the respective intensities of the two fluorescence channels, we assume a maximum intensity of 255, a mean intensity of 127 and a minimum intensity of 0 for each channel. A) The relative contribution to the PCC is shown for all possible values of **q**_*i*_, with green representing a positive and red representing a negative contribution. B) The colour that would be assigned to each voxel with intensity **q**_*i*_ according to the nMDP colourmap [11], calculated using Eq 1. Note that, at the means, there are sudden changes in the colour assigned by nMDP. C) How nMDP labels each colocalisation intensity **q**_*i*_ as colocalised or not-colocalised based on its position relative the means. A colocalisation intensity distribution commonly seen for strongly correlated fluorescence channels is shown, with darker shades indicating strong positive correlation and lighter shades indicating weaker positive correlation [3]. The areas marked in red correspond to positively correlated colocalisation that are indicated as negatively correlated when using nMDP.

Firstly, the numerator of Eq 3 can take either a positive or a negative value depending on the intensity of each fluorescence channel relative to the mean. To understand the implications of this, the relative contribution of all possible colocalisation intensities is illustrated in Fig 1A. The figure shows that the possible colocalisation intensities **q**_*i*_ are divided into two positive and two negative quadrants about the means. In Fig 1B the colour that nMDP assigns, using a colourmap, to each of these colocalisation intensities is shown. Red indicates that the underlying voxel is labelled as colocalised while blue indicates that it is labelled as not-colocalised. However, to ensure a clear interpretation of the visualisation, a linear mapping should ideally be applied to all possible colocalisation intensities. This could lead to some discontinuities in the label that is assigned around the means, as is illustrated in Fig 1C for a colocalisation intensity distribution commonly associated with two strongly correlated fluorescence channels [3]. While some follow-up studies addressed this discontinuity of the nMDP colourmap at the means by using a more gradual transition between the colocalised and not-colocalised labels [12–16]. These changes, however, alter the interpretation of the visualisation proposed by the authors of nMDP, since there is no longer a clear indication of which regions are colocalised and which are not. Furthermore, there is no straightforward way to redefine the colourmap, especially its start and end value, since nMDP can result in a wide range of numerical values.

A second disadvantage is that voxels with an intensity **q**_*i*_ close to one of the means contribute least to the PCC value while voxels for which **q**_*i*_ is far from the means contribute most. However, since only the distance from the mean and not the magnitude of the intensity is taken into account, voxels with colocalisation intensities at both the upper and lower extremes (indicated by * in Fig 1B) will be visualised with the same colour, resulting in an ambiguous visualisation. Furthermore, voxels for which **q**_*i*_ is close to one of the means will be visualised with very low intensities by the colourmap. This suppresses the majority of the voxels, which are generally close to the means, thereby possibly losing critical information in the visualisation.

Therefore, using the PCC formula directly to visualise the degree of correlation between the fluorescence colour channels has some critical drawbacks when analysing fluorescence micrographs. This is, however, not the design purpose of PCC and therefore it remains a useful way to quantify, with a single value, the degree to which the variation in intensity between the two channels can be modelled by a simple linear relationship [1]. We will use this insight to develop RACC.

### Visualising correlation using a regression line

Based on the limitations of existing visualisation methods discussed in the previous section, we develop RACC with three main objectives. Firstly, positively correlated colocalisation must be emphasized, while negatively correlated and coincidentally colocalised voxels should be suppressed. Secondly, regions of colocalised voxels with greater combined fluorescence intensities should also be emphasized, since greater fluorescence intensity is an indication of greater molecular density in the structure under investigation, and will assist in identifying molecular structures that strongly associate with one another. Thirdly, coincidentally colocalised voxels should be suppressed in the visualisation to reduce false positives.

In order to achieve these objectives we start by describing the linear relationship between the two fluorescence channel intensities. It is however not appropriate to use simple linear regression for this task, as has previously been suggested [3], since the accompanying assumptions of the ordinary least squares fit do not generally hold for colocalisation intensities [17]. Specifically, the assumption that one variable (the dependent variable) can be predicted from the other (the independent variable), and hence that the independent variable is a fixed, known constant, does not hold. Instead, both fluorescence intensities are sampled and are prone to observation errors. Therefore, we will use Deming regression, a special case of total least squares, that accounts for errors in the observations of both channel intensities [18].

For Deming regression, the regression line, or line of best fit, is described by:

$$y=\beta_{0}+\beta_{1}x$$ (4)

where *x* and *y* are the true values of the two fluorescence channel intensities and where *β*_0_ and *β*_1_ are the intercept and slope of the regression line, respectively. However, since the measured intensities $\hat{x}$ and $\hat{y}$, have associated measurement errors *ε*_*i*_ and *η*_*i*_, the true values are related to the measurements by:

$$\hat{x_{i}}=x_{i}+\eta_{i}\;,\;\;\hat{y_{i}}=y_{i}+\varepsilon_{i}$$ (5)

In Deming regression it is assumed that the ratio of the variances of these two errors is known, and is defined as:

$$\lambda =\frac{\sigma_{\eta }^{2}}{\sigma_{\varepsilon }^{2}}$$ (6)

Since the measurements of the two channel intensities are usually made under the same conditions, we assume that the variances of the two errors are similar, and hence that $\sigma_{\eta }^{2}\approx \sigma_{\varepsilon }^{2}$ and λ ≈ 1. If, however, different acquisition parameters are used to generate the fluorescence images, the true ratio of the variances of the measurement errors must be used. For the remainder of the analysis we will assume λ = 1, which corresponds to a special case of Deming regression known as orthogonal regression [19].

In order to estimate *β*_1_ we first calculate the sample mean and covariance of the random variables *X* and *Y*:

$$\bar{x}=\frac{1}{n}\sum_{i=1}^{n}x_{i}\;,\;\;\bar{y}=\frac{1}{n}\sum_{i=1}^{n}y_{i}$$ (7)

$$s_{xx}=\frac{1}{n-1}\sum_{i=1}^{n}{\left(x_{i}-\bar{x}\right)}^{2}\;,\;\;s_{yy}=\frac{1}{n-1}\sum_{i=1}^{n}{\left(y_{i}-\bar{y}\right)}^{2}$$ (8)

$$s_{xy}=\frac{1}{n-1}\sum_{i=1}^{n}\left(x_{i}-\bar{x}\right)\left(y_{i}-\bar{y}\right)$$ (9)

Now *β*_0_ and *β*_1_ are estimated as follows [18]:

$$\hat{\beta_{1}}=\frac{\lambda s_{yy}-s_{xx}\pm \sqrt{{\left(\lambda s_{yy}-s_{xx}\right)}^{2}+4\lambda s_{xy}^{2}}}{2\lambda s_{xy}}$$ (10)

$$\hat{\beta_{0}}=\bar{y}-\hat{\beta_{1}}\bar{x}$$ (11)

Because in general $\hat{\beta_{1}}$ has two solutions, the solution with the same sign as the covariance *s*_*xy*_ is conventionally chosen [20]. If the fluorescence channel intensities are positively correlated, the covariance, and therefore $\hat{\beta_{1}}$, will also be positive. However, in some rare cases the covariance can be negative, such as when the fluorescence channel intensities are not correlated or the colocalised intensities are very sparse. Our aim is to highlight voxels for which both fluorescence intensities are high, and hence we must ensure a positive slope for the regression line. Therefore, in our case, the positive solution for $\hat{\beta_{1}}$ will always be chosen.

It is standard accepted procedure in fluorescence based colocalisation analysis to remove background noise by applying thresholds to both fluorescence channels. We will refer to these thresholds as *T*_*ch*1_ and *T*_*ch*2_. In the experiments we present later, we manually determine these thresholds. However, any method of determining these thresholds may be used, for example automated thresholding [6]. A voxel is only considered colocalised if both channel intensities are above their respective thresholds. Therefore, the means and covariances in Eqs 7–9 are only calculated using the colocalisation intensities **q**_*i*_ above these thresholds.

**Fig 2:**
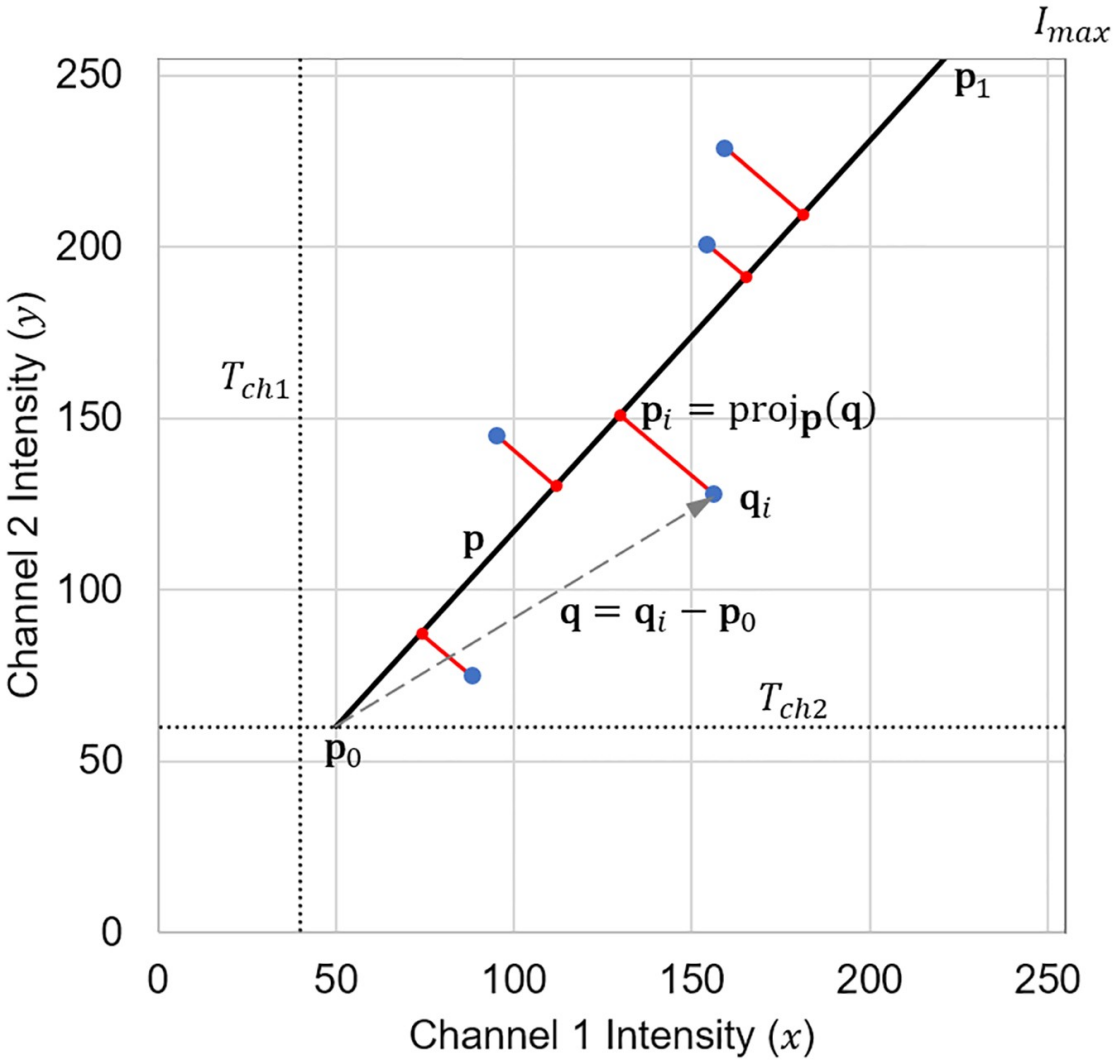
Projection of colocalised voxels on to the colourmap. A colocalisation intensity **q**_*i*_ is projected to a 1D subspace defined by **p**. A projection onto point **p**_**0**_ corresponds to a colourmap value of 0, a projection onto point **p**_1_ corresponds to a colourmap value of 1, and intermediate projections correspond to a colourmap value varying linearly between these values.

We will consider the regression line (Eq 4) as a 1D subspace onto which the colocalisation intensities **q**_*i*_ are mapped, as illustrated in Fig 2. In order to perform this mapping, it is helpful to represent the regression line as a vector **p**, that passes through two points **p**_**0**_ and **p**_1_, representing the two extremes of the 1D subspace.

The regression line must intersect one of the two lines defined by the channel thresholds. Fig 2 shows that this intersection **p**_**0**_ can be calculated as follows:

$$\begin{array}{c} p_{0}=\left(x_{p_{0}},y_{p_{0}}\right)=\{\begin{array}{cc} (T_{ch1},T_{ch1}\hat{\beta_{1}}+\hat{\beta_{0}}), & \text{if}\;\;T_{ch2}⩽T_{ch1}\hat{\beta_{1}}+\hat{\beta_{0}}\\ (\frac{T_{ch2}-\hat{\beta_{0}}}{\hat{\beta_{1}}},T_{ch2}), & \text{if}\;\;T_{ch2}>T_{ch1}\hat{\beta_{1}}+\hat{\beta_{0}} \end{array} \end{array}$$ (12)

Similarly, **p**_1_ denotes the intersection between the regression line and the maximum possible fluorescence intensity *I*_max_, and is calculated as follows:

$$\begin{array}{c} \begin{array}{c} p_{1}=\left(x_{p_{1}},y_{p_{1}}\right)=\{\begin{array}{cc} (\frac{I_{\text{max}}-\hat{\beta_{0}}}{\hat{\beta_{1}}},I_{\text{max}}), & \text{if}\;\;\hat{\beta_{0}}⩾I_{\text{max}}\left(1-\hat{\beta_{1}}\right)\\ (I_{\text{max}},I_{\text{max}}\hat{\beta_{1}}+\hat{\beta_{0}}), & \text{if}\;\;\hat{\beta_{0}}<I_{\text{max}}\left(1-\hat{\beta_{1}}\right) \end{array} \end{array} \end{array}$$ (13)

The colocalisation intensity of each voxel **q**_*i*_ can now be projected perpendicularly onto the regression line **p**, resulting in a point **p**_*i*_. To achieve this projection we define the vector from **p**_**0**_ to **q**_*i*_ as **q** (Fig 2) from which it follows that:

$$p_{i}=\left(x_{p_{i}},y_{p_{i}}\right)={\text{proj}}_{p}\left(q\right)=\frac{q\cdot p}{p\cdot p}p+p_{0}$$ (14)

This projection is used to assign a colourmap value *C*_*i*_ between 0 and 1 based on the linear position of **p**_*i*_ along **p**. This value is in turn used to assign a colour to the *i*^th^ voxel by means of a colourmap. Note that if **p**_1_ does not correspond exactly to the intensity (*I*_max_, *I*_max_), the perpendicular projection of the colocalisation intensity **q**_*i*_ may result in some points **p**_*i*_ positioned beyond the maximum of the colourmap **p**_1_. The colourmap value *C*_*i*_ of such voxels are assigned a colourmap value of 1. Similarly, if **p**_**0**_ does not correspond exactly to the intensity (*T*_*ch*1_, *T*_*ch*2_), **p**_*i*_ may lie below **p**_**0**_. These voxels are assigned a colourmap value of 0. Therefore, *C*_*i*_ can be calculated as follows:

$$\begin{array}{c} \begin{array}{c} C_{i}=\{\begin{array}{cc} 0, & \text{if}\;\;\;x_{p_{i}}\leq x_{p_{0}}\\ \frac{x_{p_{i}}-x_{p_{0}}}{x_{p_{1}}-x_{p_{0}}}, & \text{if}\;\;\;x_{p_{0}}<x_{p_{i}}<x_{p_{1}}\\ 1, & \text{if}\;\;\;x_{p_{i}}\geq x_{p_{1}} \end{array} \end{array} \end{array}$$ (15)

**Fig 3:**
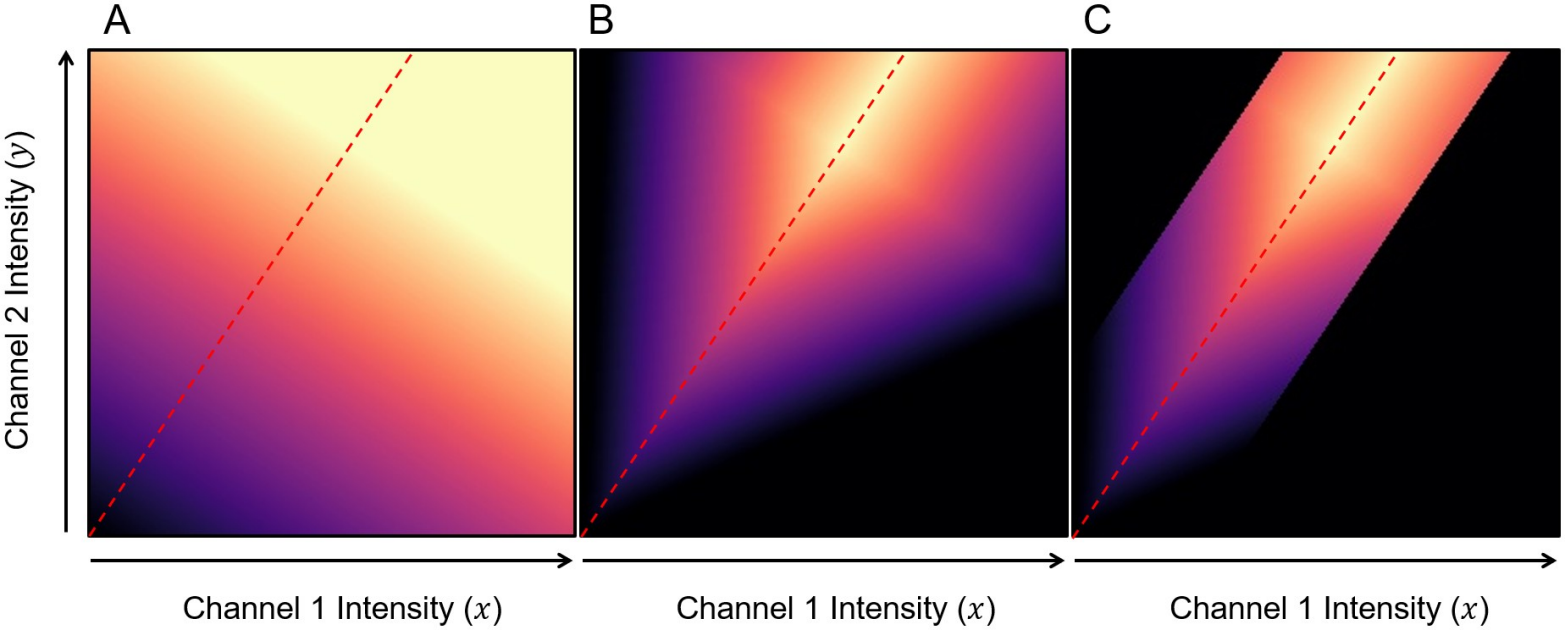
The effect of the colour mapping model on possible colocalisation intensities. Using Fig 2 as a reference, we consider a specific regression line, shown as a red dashed line, with $\hat{\beta_{1}}=1.5$ and $\hat{\beta_{0}}=0$ for illustration. We visualise the corresponding colourmap value (*C*_*i*_) calculated for each possible colocalisation intensity **q**_*i*_ = (*x*_*i*_, *y*_*i*_) using the Magma colourmap, where black corresponds to *C*_*i*_ = 0 and bright yellow corresponds to *C*_*i*_ = 1. For clarity, both thresholds *T*_*ch*1_ and *T*_*ch*2_ are zero in this example. A: The colourmap values *C*_*i*_ using Eq 15. B: The colourmap values $C_{i}^{\prime }$ using Eq 17 with *θ* = 60°. C: The final colourmap values *C*_*i*_ using Eq 19 with *d*_*t*_ = 0.2 and $x_{p_{\text{max}}}=0.8I_{\text{max}}$.

To understand the effect of Eq 15 on the voxels in a sample, we plot the colourmap intensity *C*_*i*_ that would be used for every possible colocalisation intensity **q**_*i*_, for a particular regression line, in Fig 3A using the perceptually uniform Magma colourmap. From this it becomes clear that voxels that have high fluorescence intensities for both *x* and *y* are assigned a high value of *C*_*i*_, while lower fluorescence intensities are assigned lower values of *C*_*i*_. Eq 15 therefore satisfies our second objective of highlighting voxels with greater combined fluorescence intensities. However, it does not yet satisfy our first and third objectives, since the model does not suppress voxels that are not positively correlated. Instead, it assigns the same colourmap value to all colocalisation intensities that lie on a line perpendicular to the regression line.

To fulfill the first objective, we recall that the regression line represents correlation in the data and therefore we seek to suppress voxels for which **q**_*i*_ is far from the regression line. To achieve this we calculate the normalized perpendicular distance *d* from **q**_*i*_ to the regression line **p** and adjust the colourmap value accordingly. This normalized distance *d* is given as:

$$d=\frac{\left|q_{i}-p_{i}\right|}{I_{\text{max}}}=\frac{\sqrt{{\left(x_{i}-x_{p_{i}}\right)}^{2}+{\left(y_{i}-y_{p_{i}}\right)}^{2}}}{I_{\text{max}}}$$ (16)

The normalization by *I*_max_ ensures that 0 ≤ *d* ≤ 1.

**Fig 4:**
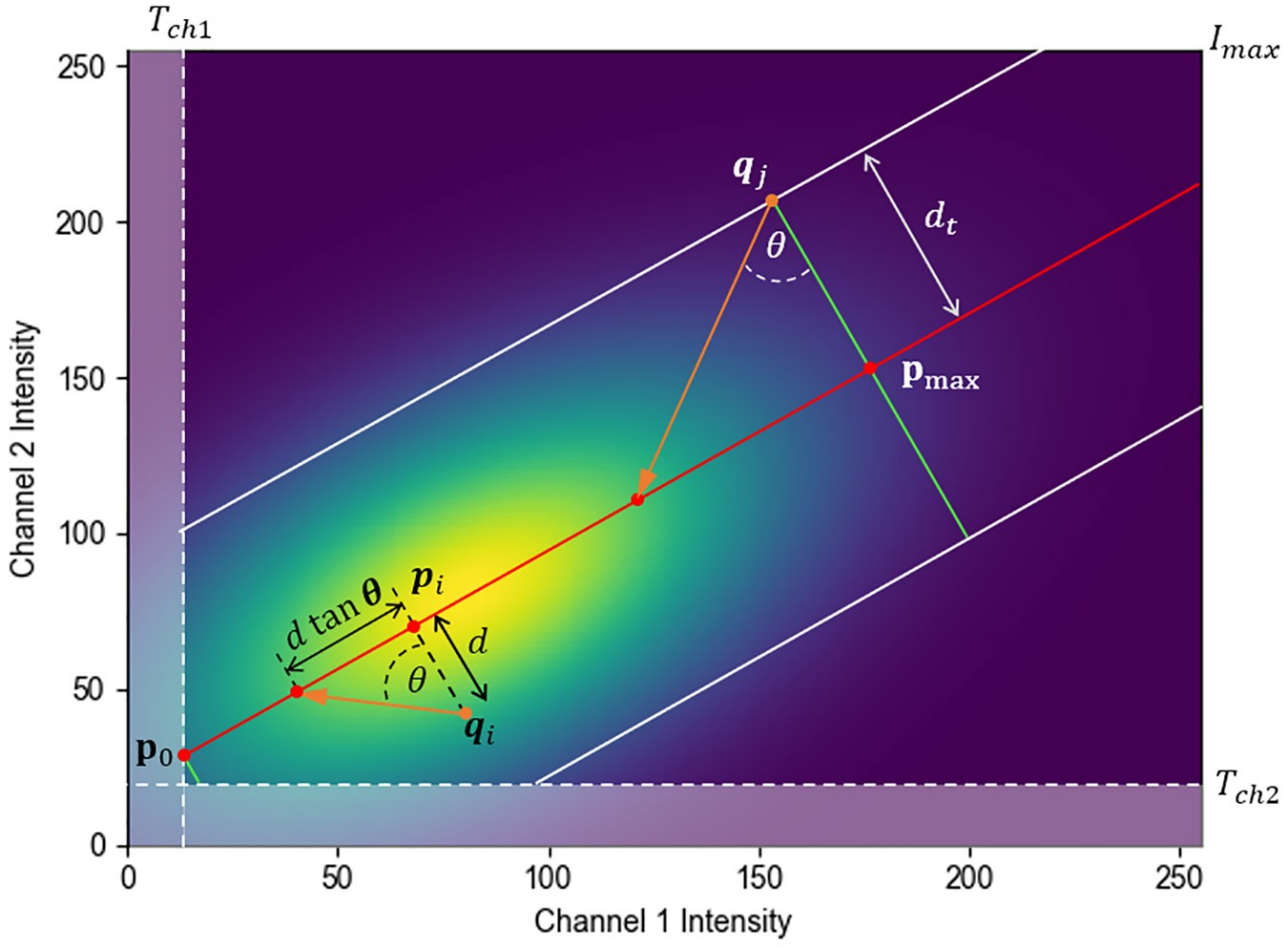
Illustration of the effect of the colourmap thresholds using a synthetically generated scatter plot, where the colourmap brightness indicates the frequency of voxels with a given colocalisation intensity. The regression line is shown in red, with two example intensity pairs, **q**_*i*_ and **q**_*j*_, shown in orange. A penalization factor *θ* is applied when projecting both **q**_*i*_ and **q**_*j*_ onto the regression line. Only the sample data above the channel thresholds, *T*_*ch*1_ and *T*_*ch*2_, are considered when determining the distance threshold *d*_*t*_, shown in white. Any colocalisation intensities **q**_*i*_ beyond the maximum point **p**_max_ are assigned a colourmap value of 1. Similarly any **q**_*i*_ below the minimum point **p**_0_ are assigned a colourmap value of 0. These thresholds are indicated with two green lines at the respective points. Both *d*_*t*_ and **p**_max_ are determined independently to include 99% of the data, thereby excluding outliers from the visualisation. The final colourmap value *C*_*i*_ varies linearly between 0 to 1 from **p**_0_ to **p**_max_.

The colourmap value *C*_*i*_ originally calculated by Eq 15 is then attenuated linearly with the distance *d*. This is achieved by projecting each colocalisation intensity *q*_*i*_ onto the regression line at an angle *θ* to the perpendicular, as illustrated in Fig 4. The value of this *penalization factor* *θ* therefore determines the extent to which the colourmap value *C*_*i*_ is attenuated as *d* increases. Attenuation is achieved using the following equation, with *C*_*i*_ calculated in Eq 15.

$$\begin{array}{c} \begin{array}{c} C_{i}^{\prime }=\{\begin{array}{cc} C_{i}-d\;\text{tan}\left(\theta \right), & \text{if}\;\;C_{i}>d\;\text{tan}\left(\theta \right)\\ 0, & \text{if}\;\;C_{i}\leq d\;\text{tan}\left(\theta \right) \end{array} \end{array} \end{array}$$ (17)

In Eq 17 it has been assumed that 0° ≤ *θ* < 90°. A penalization factor of 0° corresponds to no attenuation, and hence $C_{i}^{\prime }\equiv C_{i}$, while factors close to but excluding 90° correspond to maximum attenuation, effectively making *C*_*i*_ = 0, and completely suppressing all voxels for which **q**_*i*_ is not positioned precisely on the regression line. The effect of Eq 17 on all possible colocalisation intensities **q**_*i*_ is visualised in Fig 3B for *θ* = 60°. The penalization factor *θ* is the only adjustable parameter in the RACC method and can be determined interactively by varying it over the range [0, 90). This allows the investigator to adjust the degree to which the voxels that show weak correlation between the fluorescence colours should be suppressed. Choosing a low penalization factor (low value of *θ*) can cause the visualization to become saturated with high colourmap values, while choosing a high penalization factor (high value of *θ*) can lead to saturation with low colourmap values. The best choice will differ between samples. Once this value is found for a particular sample or condition, similar samples can subsequently be visualized with the same value to allow for the best and standardised comparison. Attempts can be made to automatically determine the value of the penalization factor, however for the samples we analysed in this paper we found that a value of *θ* = 45° often produces favourable results.

To meet our third stated objective of suppressing coincidental colocalised voxels from the visualisation, we introduce a distance threshold *d*_*t*_ (Fig 4) which we use to remove outliers around the regression line. Only voxels for which the distance *d* between the colocalisation intensity **q**_*i*_ and the regression line is below this threshold are visualised. The threshold *d*_*t*_ is determined automatically to include 99% of all voxels with intensities above the channel thresholds *T*_*ch*1_ and *T*_*ch*2_. Assuming a normal distribution of the intensities, this corresponds to approximately three standard deviations from the mean. The effect of applying a distance threshold *d*_*t*_ = 0.2 is illustrated in Fig 3C. The corresponding colourmap value is calculated using Eq 18:

$$C_{i}^{\prime \prime }=\{\begin{array}{cc} C_{i}^{\prime }, & \text{if}\;\;d\leq d_{t}\\ 0, & \text{if}\;\;d>d_{t} \end{array}$$ (18)

Finally, in scenarios where most voxels have low intensities for both channels, for example due to low intensity in the fluorescence microscopy acquisition parameters, there will be an ineffective utilization of the colourmap spectrum and an associated limited variation in the visualisation. To address this, we define a point $p_{\text{max}}=\left(x_{p_{\text{max}}},y_{p_{\text{max}}}\right)$ on the regression line that indicates the maximum represented intensities for the image. Any projected point **p**_*i*_ beyond **p**_max_ is assigned a colourmap value of 1. The point **p**_max_ is calculated to include 99% of all sample data above the channel thresholds. Since all colocalisation intensities are projected perpendicularly onto the regression line, the imposition of **p**_max_ has the effect of saturating the colourmap for voxels whose projected intensities fall beyond **p**_max_, indicated in green in Fig 4. This makes more of the colourmap available for the representation of lower colocalisation intensities, thereby enabling clearer data interpretation. This re-scaling of the applied colourmap is achieved by replacing **p**_1_ with **p**_max_ in Eq 15. The researcher may, however, choose not to apply this scaling in which case **p**_1_ is retained.

Given **q**_*i*_ and *θ* and obtaining the parameters **p**_0_, **p**_*i*_ and *d* from Eqs 12, 14 and 16, Eq 15 can now be re-written in its final form as follows:

$$\begin{array}{c} \begin{array}{c} C_{i}=\{\begin{array}{cc} 0, & \text{if}\;\;\;x_{p_{i}}\leq d\left(x_{p_{\text{max}}}-x_{p_{0}}\right)\text{tan}\left(\theta \right)+x_{p_{0}}\;\;\text{or}\;\;d>d_{t}\\ \frac{x_{p_{i}}-x_{p_{0}}}{x_{p_{\text{max}}}-x_{p_{0}}}-d\;\text{tan}\left(\theta \right), & \text{if}\;\;\;d\left(x_{p_{\text{max}}}-x_{p_{0}}\right)\text{tan}\left(\theta \right)+x_{p_{0}}<x_{p_{i}}<x_{p_{\text{max}}}\\ 1-d\;\text{tan}(\theta ), & \text{if}\;\;\;x_{p_{i}}\geq x_{p_{\text{max}}} \end{array} \end{array} \end{array}$$ (19)

The result of Eq 19 is a colourmap value *C*_*i*_ between 0 and 1 for any pair of measured fluorescence intensities **q**_*i*_ = (*x*_*i*_, *y*_*i*_).

## Results and discussion

With the aim of improving the 3D spatial visualisation of colocalisation in biological samples by incorporating both the correlation and the underlying fluorescence channel intensity in the visualisation, we developed the regression adjusted colocalisation colour mapping (RACC).

In the following, we will apply and validate the RACC algorithm by visualising colocalisation in both synthetically generated data as well as in three distinct biological samples. This is intended to demonstrate the advantages offered by RACC.

In the first biological sample we visualise the colocalisation between *α*/*β* tubulin and acetylated tubulin. In the second and third samples we visualise the colocalisation between two organelles and between an organelle and tubulin respectively. In the latter cases, in particular, we investigate the fused state between lysosomes and autophagosomes as well as between autophagosomes and the tubulin network, both aspects of biomedical importance. Due to the small size of the organelles and the fineness of the filamentous tubulin structures, it is often challenging to accurately investigate the degree of colocalisation between them. These structures were therefore deliberately chosen to demonstrate the performance of RACC in challenging scenarios. The synthetic data was designed to mimic and better demonstrate aspects of these biological samples.

We will compare RACC (Eq 19) to the nMDP (Eq 1) throughout, since nMDP is currently the most widely used method of spatially visualising the correlation of the colocalised voxels using a colourmap. For both RACC and nMDP, all parameters were calculated over the entire 3D z-stack. For purposes of comparison, we used manual intensity thresholding to remove the background intensities for both methods. These thresholds are shown in the scatter plots accompanying the figures. Part of the design of RACC is the automatic adjustment of the maximum colourmap value to ensure the optimal use of the available spectrum. For nMDP, however, it is standard practice to fix the minimum and maximum values to -1.0 and 1.0, respectively [11]. In our experience, this does not always generalize optimally, especially since nMDP can produce values beyond these limits. Therefore, to ensure the best visualisation for each image set, we adjusted the minimum and maximum values of the nMDP colourmap manually. These values are indicated on the nMDP colourbars in the figures. Similarly, the penalization factor *θ* that was used for each sample is shown on the colourbar.

### Visualising synthetic data

**Fig 5:**
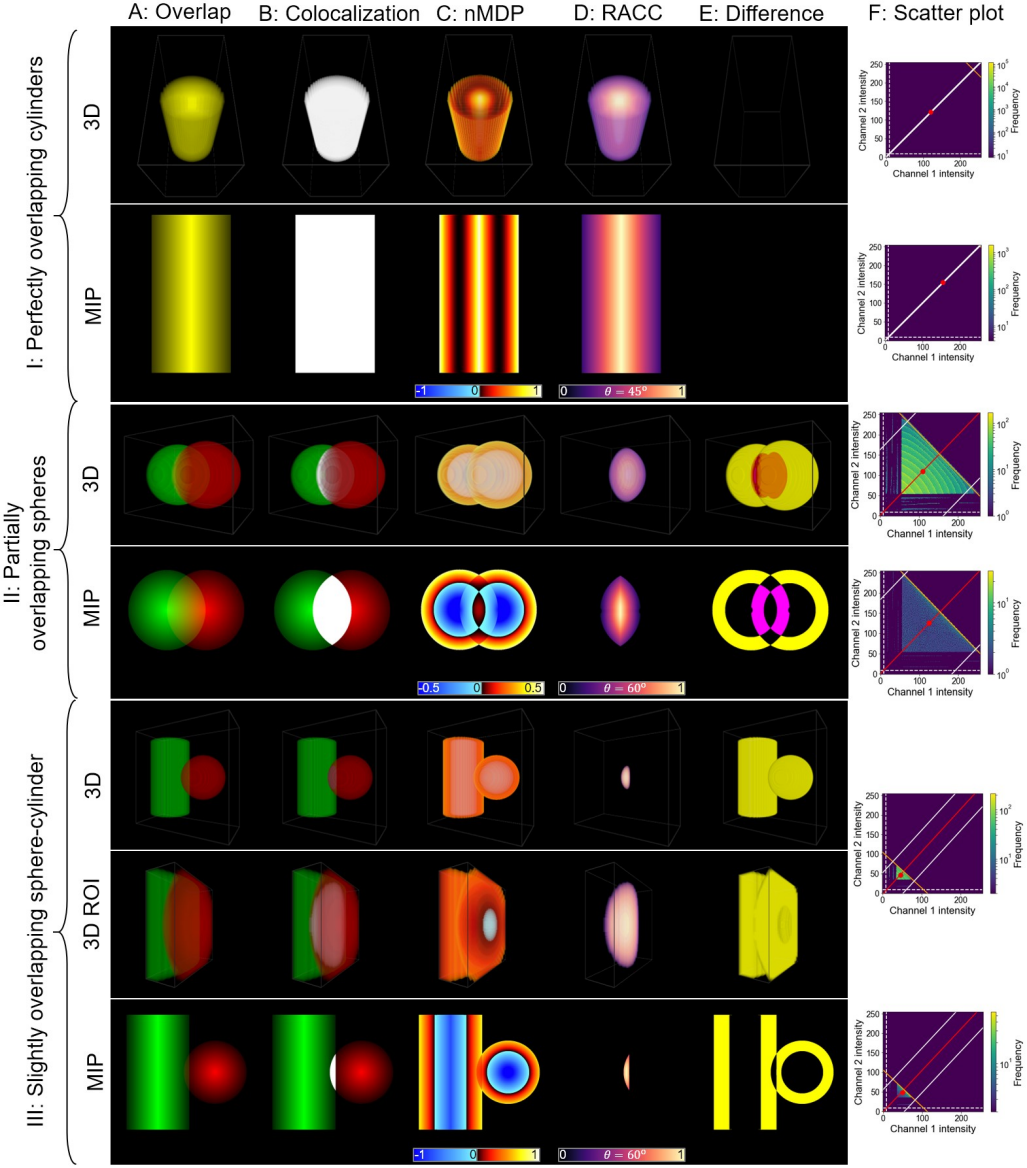
Visualisation of synthetic data using both 3D images and maximum intensity projections (MIP). A: The overlapping fluorescence channel intensities. B: All voxels above the colocalisation thresholds and therefore considered colocalised are overlaid in white. C: The result of applying the nMDP. D: The result of applying RACC. E: The difference between RACC and the nMDP. Magenta represents areas which nMDP considers not-colocalised but that RACC considers colocalised, while yellow represents areas that nMDP considers colocalised but RACC does not. F: Scatter plots for the 3D and MIP datasets, showing the frequency of each colocalisation intensity. The regression line calculated by RACC is shown in red. The maximum and distance thresholds for RACC are shown in orange and white, respectively. The red marker represents the per-channel average intensities used by the nMDP calculations and around which the four quadrants are separated (see Fig 1).

In order to better understand how RACC visualises colocalisation in biological samples we generated synthetic data that is illustrative of some aspects of the biological samples that we investigate. We consider three sets of synthetic data, shown in section I, II and III of Fig 5. Section I shows two perfectly overlapping cylinders of the same size. These model the overlap between *α*/*β* tubulin and acetylated tubulin that will be seen in the biological samples. Section II shows two partially overlapping spheres, and are an idealisation of autophagasome-lysosome fusion. Finally, section III shows a slightly overlapping cylinder and sphere, representing autophagasome-tubulin interaction. This scenario is similar to the start of autophagasome-lysosome fusion which we modelled using two slightly overlapping spherical structures. For all synthetic images, the highest colour intensity occurs at the center of the spheres and cylinders and decreases towards the surface of these structures. This mirrors the observed fluorescence intensities in the biological samples. Furthermore, in the case of the synthetic data, it is often insightful to consider the maximum intensity projection (MIP), which provides an internal perspective of the 3D datasets. This is useful because most of the variation in fluorescence intensity occurs inside the volume and not at the surface.

#### Technical discussion

Fig 5A shows the colocalisation between red and green channels as yellow, and Fig 5B shows the same colocalised voxels in white. These are the most common ways of visualising colocalisation. From Fig 5B it is expected that a colocalisation colourmap method should only assign a colourmap value to the voxels in white. This value must vary with the channel intensities of the colocalised voxels in a manner similar to that seen in Fig 5A.

When considering Fig 5C, we recall that nMDP visualises colocalised voxels in shades of red to yellow, while voxels that are not-colocalised are visualised in shades of blue (see Fig 1B). By considering sections II and III of Fig 5C, it is clear that, the nMDP assigns a colourmap value to all voxels for which either fluorescence channel is present, and not only to those that would normally be considered colocalised (indicated as white in Fig 5B). Since it is common for the channel means (indicated by a red dot in Fig 5F) to be greater than the intersection point of the two channel thresholds, lower intensity voxels are not removed from the visualisation. This leads some of these voxels to be assigned colocalised colourmap values according to Fig 1B, which could misrepresent the data. Fig 5D on the other hand, shows that RACC only assigns colours to voxels that are considered colocalised and does not visualise voxels that are not colocalised. This occurs because RACC is only calculated for voxels for which *both* fluorescence intensities are above the channel thresholds, whereas nMDP is calculated when a single fluorescence channel is above the respective channel threshold. Fig 5E visualises this difference by showing regions that are labelled as colocalised by nMDP but not by RACC in yellow, and regions for which the converse is true in magenta. The same information is also presented quantitatively in S1 Table which shows the percentage of the total voxels that were classified differently by RACC and nMDP.

In the case of the perfectly overlapping cylinders (Fig 5 section I), both fluorescence channels are perfectly correlated. Initially the nMDP decreases from a high intensity at the center of the cylinder to its periphery. However, at lower fluorescence intensities an increase is again observed, leading to an unintuitive interpretation of the correlation in the colocalisation. This increase is due to the nMDP calculation only considering the deviation from the mean and not the magnitude of the intensity, as shown in Fig 1B. RACC shows a linear decrease from the high fluorescence intensities at the center of the cylinder to the lower intensities at its periphery, thereby highlighting volumes with higher fluorescence intensity, which typically correspond to a higher concentration of the fluorescent label. Note that since the *x* and *y* of all colocalisation intensities are the same and form a line on the scatter plot in Fig 5F, the penalization factor *θ* has no effect on the visualisation.

When considering the partially overlapping spheres (Fig 5 section II), the fluorescence intensities at the center of the overlapping volume are strongly correlated, with correlation decreasing towards the edges of the overlapping region. This can be easily discerned from the RACC visualisation in column F. Notice also that the left and right sides of the RACC visualisation are slightly clipped due to the distance threshold (Eq 18) which removes outliers. Furthermore, since the maximum point is calculated to include 99% of the voxels (indicated by the orange line in Fig 5F), the entire colourmap spectrum is used for the visualisation. On the other hand, the nMDP visualisation is more ambiguous, since the region near the surface of the spheres appears to contain the majority of the colocalised voxels, while in fact there is no colocalisation in that region as indicated by column B. Furthermore, the nMDP does not correctly show the entire overlapping volume as colocalised, with the overlapping volume further from the center being shown as not-colocalised.

When considering the slightly overlapping sphere and cylinder (Fig 5 section III), there is only a small region in which colocalisation occurs. Since the intensities of the two channels are similar in the colocalised region, they should be fairly well correlated. This aspect is intuitively reflected when using RACC, where regions of similar fluorescence intensity in the center of the overlapping region are highlighted. As in the case of the partially overlapping spheres, regions of lower channel intensity near the surface are again erroneously labelled as colocalised by the nMDP. Moreover, the nMDP does not visualise the overlapping volume noticeably different from the surrounding structure. This leads to a visualisation that could be difficult to interpret.

### Visualising biological data

The nMDP has been and continues to be used in many studies in the life sciences, where the precise spatial analysis of colocalisation is of particular relevance. These include the assessment of molecular interactions at the neuromuscular junction [12], the analysis of vesicular structures part of the endosomal compartment [21, 22], the characterization of filamentous actin or tubulin network structures [13, 15] and the localization and assessment of the degree of protein interaction at the subcellular level [14, 16].

In most of these studies, the quantification of colocalisation based on a colourmap is limited and conclusions are based primarily on the comparison of colocalisation metrics such as the PCC. RACC may offer new perspectives, by restricting the analysis to the colocalised voxels and by allowing a more robust identification of the intensity *correlation* at colocalised voxels.

In the following we apply both nMDP and RACC to a subset of biological samples with similar complexity and compare the results.

#### Cell culture and transfections

Mouse embryonic fibroblasts (MEFs) as well as stably expressing GFP-LC3 MEF’s were supplemented with Dulbecco’s Modified Eagles Medium (DMEM), 1% penicillin/streptomycin (PenStrep) (Life Technologies, 41965062 and 15140122) and 10% foetal bovine serum (FBS) (Scientific Group, BC/50615-HI) and incubated using a humidified incubator (SL SHEL LAB CO2 Humidified Incubator) in the presence of 5% CO_2_ at 37°C. Where applicable, cells were micropatterned, as previously shown [23]. Cells were seeded in either an 8-chamber cover slip-based dish (Nunc, Lab-Tek, 155411) or a micropatterned slide for subsequent experiments.

#### Immunofluorescence and super resolution structured illumination microscopy (SR-SIM)

In brief, MEF cells were fixed in 4% formaldehyde (Sigma-Aldrich, USA), washed with phosphate buffered saline (PBS) and permeabilized using 0.2% Triton-x, followed by a blocking step using 1% bovine serum albumin (BSA). Thereafter, cells were incubated at 4°C overnight in primary antibodies against *α*/*β* tubulin (Cell Signaling, #2148), acetylated tubulin (SC-23950, Santa Cruz) and LysoTracker red (Life Technologies, #L-7528), followed by staining with secondary antibodies Alexa-488 and Alexa-561. SR-SIM and confocal micrographs were acquired using the ELYRA PS.1 station (Carl Zeiss Microimaging; Germany). Thin (0.1 *μ*m) Z-stacks of high-resolution image frames were collected in 3 rotations, followed by reconstruction using ZEN software (black edition, 2011, version 7.04.287) based on a structured illumination algorithm [24].

#### Analysis of *α*/*β* tubulin and acetylated tubulin overlap

The tubulin network in a cell is characterized by dynamic modifications such as acetylation, which impacts its properties such as stiffness and function. Here, we assess the degree of acetylation relative to the entire tubulin network through colocalisation analysis. Since microtubulues are delicate filamentous structures, their overlap is very challenging to analyse.

**Fig 6:**
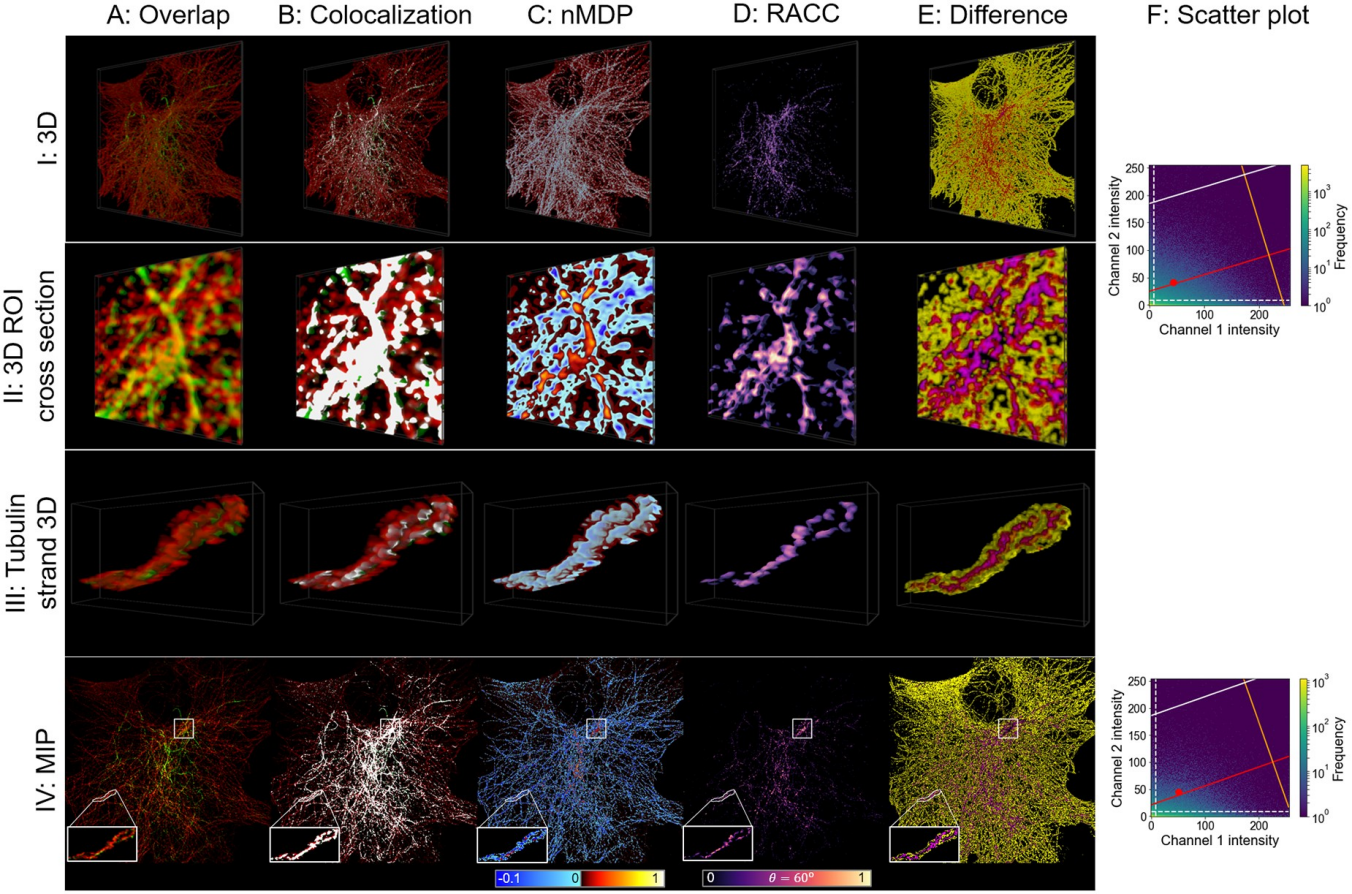
*α*/*β* tubulin (red) and acetylated tubulin (green) visualisation. The structure of the figure is similar to that of Fig 5, but in this case biological samples and not synthetic data are considered.

In order to analyse colocalisation, we make specific ROI selections at the cell’s periphery (row II in Fig 6) as well as a cross section along the z-axis in the perinuclear region (row III in Fig 6). This cross section was introduced so that the colocalisation inside the tubulin can also be visualised, where the correlation between the fluorescence channels is the strongest. In this way, we are able to analyse colocalisation with precision in regions with both high and low fluorescence channel intensity. Where these selections are located is shown on the MIP in row IV of Fig 6.

In row II of the nMDP visualisation in Fig 6C, the central area is clearly highlighted as being colocalised. However, since a colourmap value is assigned to all voxels for which a fluorescence channel is present, it is not possible to clearly discern the extent of this colocalisation. RACC similarly highlights the central region as colocalised (row II in Fig 6D). However, it also indicates that there is a greater extent of colocalisation in this region. Regions of lower intensity and with a weaker correlation between fluorescence channels are retained when using RACC and are shown as colocalised in darker hues of blue, whereas nMDP reports these regions as not colocalised. Furthermore, nMDP falsely demarcates several regions around edges of the sample as being colocalised, indicated by yellow in Fig 6E, which leads to a visualisation that detracts from the truly colocalised regions.

A prominent tubulin strand was isolated from the sample in row III of Fig 6D. Here the nMDP indicates no colocalisation, while RACC is able to indicate this volume as colocalised and allows one to discern the varying degree of correlation.

#### Analysis of autophagasome and lysosome fusion

**Fig 7:**
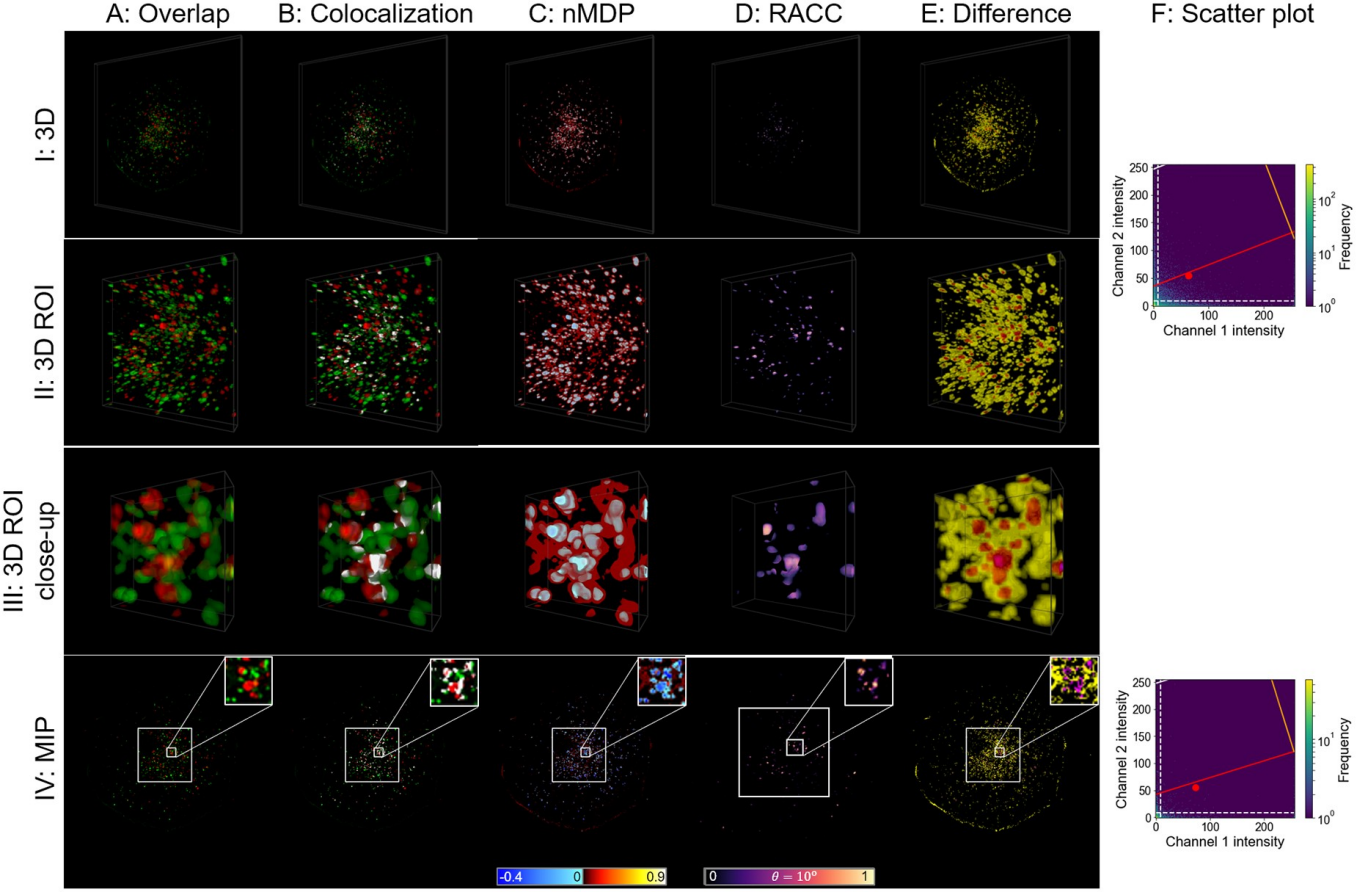
Autophagasome (green) and lysosome (red) fusion visualisation. The structure of the figure is similar to that of Fig 5, but in this case biological samples and not synthetic data are considered.

Protein degradation though macroautophagy plays a critical role in cellular homeostasis, metabolism and disease [25]. It is characterized by the delivery of autophagosomes to lysosomes, where, upon fusion, an autolysosome is formed, and hydrolytic degradation takes place. To better understand autophagy function and dysfunction, it is of major interest to discern the contribution of the pathway intermediates to the total intracellular vesicle pool size, i.e. autophagosomes, autolysosomes and lysosomes [23]. Visualising the fusion zone between autophagosomes and lysosomes is hence of critical importance, and will be used here as a second example to compare RACC and nMDP (Fig 7).

Due to the small size of these organelles, it is challenging to visualise the degree of fusion that has taken place. Note that this fusion is what has been modelled in the synthetic data by means of partially overlapping spheres. We recall from the synthetic data that the nMDP erroneously indicated regions near the surface of the spheres as colocalised. A similar observation is made in the case of the here described autophagosome/lysosome fusion in Fig 7C. This may detract from the true interaction between the organelles and hence from the correct interpretation of the colocalisation data. This is also reflected in the substantial difference between nMDP and RACC shown in Fig 7E.

RACC shows only the colocalised voxels, which allows the colocalised regions with a strong correlation of the fluorescence channels (high intensities of the RACC colourmap) as well as regions with lower correlation (darker blue regions) to be clearly discerned. This is best seen in rows II and III of Fig 7D. By isolating a small region of the cell, shown in row III, RACC reveals which of these colocalisations indicates an almost complete fusion between the organelles and hence the extent of autophagy progression.

#### Analysis of autophagasome and tubulin interaction

Autophagosomal transport is facilitated by the microtubule network [26]. Hence, the interaction between these two structures and its accurate visualisation is of major interest.

**Fig 8:**
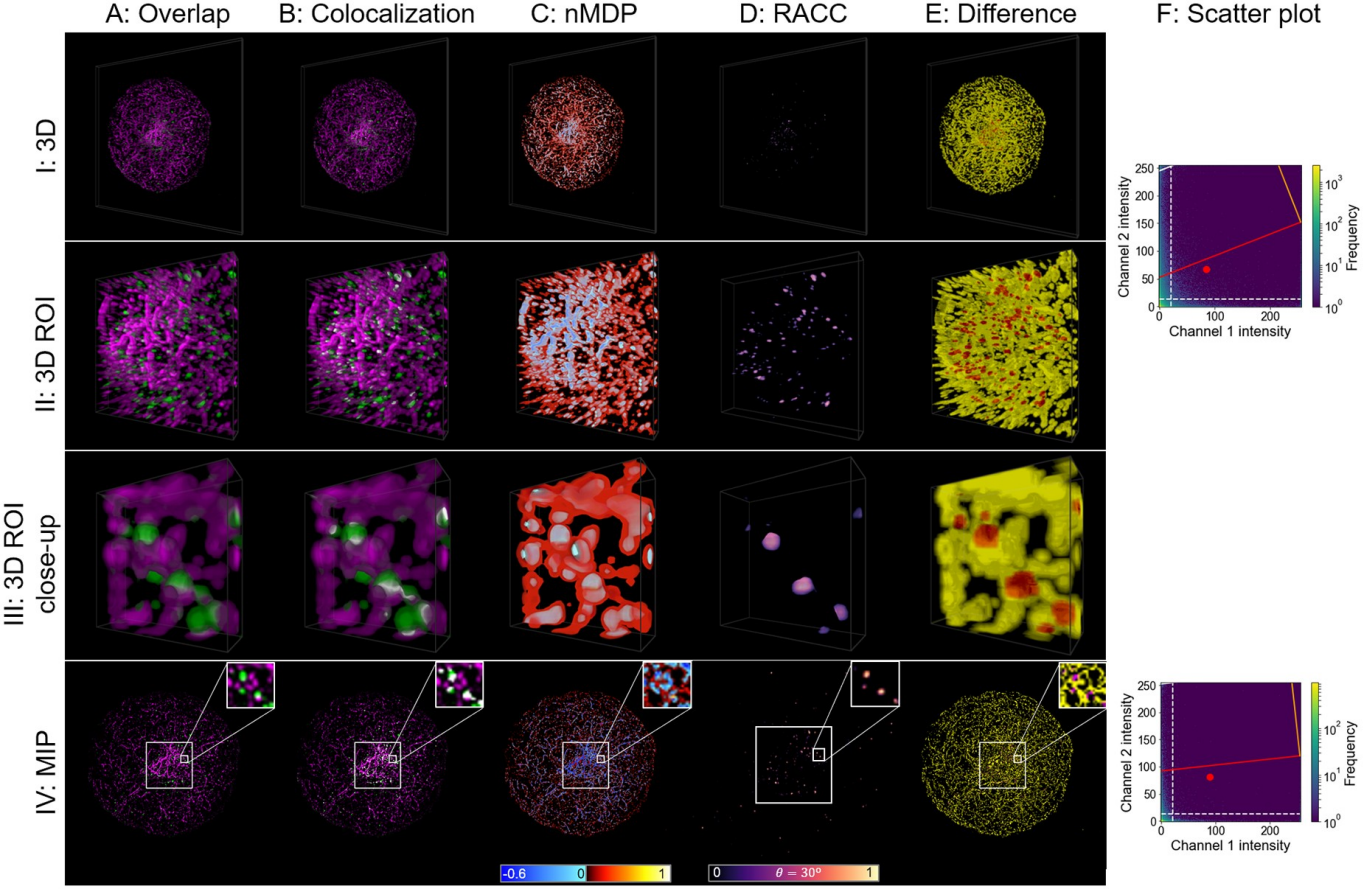
Autophagasome (green) and tubulin (magenta) visualisation. The structure of the figure is similar to that of Fig 5, but in this case biological samples and not synthetic data are considered.

In Fig 8 we show such autophagasome and tubulin interaction. Since the autophagasomes are transported along the tubulin network, there is very little overlap between them and consequently very few colocalised voxels. We therefore assess two ROIs in order to investigate the interaction in more detail. These ROIs are shown in rows II and III of Fig 8 and their location within the sample as a whole is shown on the MIP in row IV.

As for autophagasomes and lysosomes (Fig 7C), the nMDP in Fig 8C does not clearly indicate the correlation of the colocalisation between the fluorescence channels. This is mainly due to the incorrect identification of voxels at the edge of the structures as colocalised. A similar result was also observed for the synthetic data consisting of partially overlapping spheres.

The magenta areas in row III of Fig 8E, which appear red due to the yellow surrounding voxels, also match the structures that RACC identifies as colocalised in Fig 8D. This indicates that nMDP is not suitable to precisely and correctly visualise the truly colocalised regions, shown in white in Fig 8B. This can be ascribed to the lack of correlation between the fluorescence channels, as can be seen in the scatter plots. As a result, most of the high intensity voxels, located inside the structure, are labelled as not-colocalised (blue). The lower intensity voxels, which surround the structure, are labelled as colocalised (red). However, there is little true colocalisation in this sample, and the weak true colocalisation signal localises in the higher-intensity region. nMDP is not able to adequately compensate for this imbalance, contributing to the observed differences. The percentage of the total voxels that were classified differently by RACC and nMDP is shown in S1 Table.

Since RACC shows only colocalised voxels, by considering it in conjunction with the overlapping fluorescence channels (Fig 8A and 8B), a better understanding of the interaction between the autophagasome and tubulin can be obtained. The qualitative extent of these colocalisation events can also be determined more intuitively.

### Discussion

Although methods to visualise the correlation between two colocalised fluorescence channels in a qualitative way exist, challenges remain when interpreting these visualisations, due to ambiguity introduced into the visualisation due to not properly considering the colocalisation of the fluorescence channels. Here we have demonstrated how RACC can offer an improved interpretability of the nature of the colocalisation in different regions of a sample. The synthetic and biological sample data used serve to indicate that RACC can be applied to a variety of colocalisation investigations, including samples that are challenging to analyse. As the scatter plots that accompany the figures show, RACC enables the visualisation of data even when no clear correlation between the fluorescence channel intensities can be discerned.

Even though RACC can be used as a stand-alone analysis tool, it may be more powerful when utilized in conjunction with the popular overlapping fluorescence channel visualisation (sub-figures A and B in Figs 5–8), since it contextualizes the colocalisation within the surrounding cell structure.

The RACC parameters that were used when visualising the samples presented in this paper were calculated over the sample as a whole. Since the ROIs that we isolated were only intended to show the differences between nMDP and RACC in greater detail and not to make biological conclusions, we chose not to recalculate the RACC parameters for individual ROIs. However, such recalculations based on careful ROI selections are strongly advised when region-specific analysis is required, since each subcellular region can have different colocalisation correlation distributions, and consequently different regression lines. Furthermore, care should be taken to minimize additional factors that could influence the fluorescence channel intensities, such as the relative quantum yield of fluorophores, the collection efficiency of the microscope and the detector gain [27].

An implementation of RACC, allowing the visualisation of both 2D and 3D samples, is included as S1 File and is also available for download at https://rensutheart.github.io/RACC.html.

## Conclusion

Visual colocalisation analysis in fluorescence-based microscopy is a very important method used by biologists to gain an understanding of the association between molecular structures within a cell. However, existing methods of colocalisation visualisation that qualitatively visualise the spatial distribution of the correlation between the fluorescence channels in a sample have several limitations such which could lead them to produce ambiguous visualisations in certain situations, thereby reducing their usefulness.

To overcome these limitations, we present RACC (regression adjusted colocalisation colour mapping), a novel biological visual analysis method that offers improved spatial visualisation of colocalisation in fluorescence-based micrographs using a colourmap. RACC is designed to meet three objectives: firstly to highlight regions within a cell that have a strong positive correlation between two fluorescence channels, secondly to highlight colocalised regions that have greater combined fluorescence intensities and thirdly to suppress coincidentally colocalised voxels from the visualisation. These objectives were achieved by applying Deming regression in conjunction with a geometric projection of the fluorescence intensities while using automatically calculated thresholds to remove outliers.

By visualising both synthetic data and three biological samples, RACC was compared with a prominent existing method used to visualise the channel intensity correlation of colocalised volumes using a colourmap. These visualisations demonstrated how RACC can offer an improved understanding of the nature of the colocalisation within a sample by visualising only truly colocalised regions while offering a qualitative measure of the correlation between fluorescence channels in different regions of a sample. This positions RACC as an attractive colocalisation analysis tool especially in scenarios characterized by complex structure and biology.

It is hoped that the application of RACC in fluorescence microscopy will enable better discrimination of colocalisation events and in turn assist future biological investigations where colocalisation analysis is central.

## Supporting information

S1 Table Percentage of the total voxels classified differently by RACC and nMDP. (XLS)

S1 File RACC software utility with example images. (ZIP)
