# Supplementary material for: Regression adjusted colocalisation colour mapping (RACC): A novel biological visual analysis method for qualitative colocalisation analysis of 3D fluorescence micrographs
Source: PLoS One. 2019 Nov 11;14(11):e0225141. doi: 10.1371/journal.pone.0225141 (PMC6844467; doi:10.1371/journal.pone.0225141)
Supplement: S1 File — (ZIP) [file pone.0225141.s002.zip › RACC_v0.8/RACC_website.html]

RACC | Rensu Theart’s blog

Skip to the content.


# Regression adjusted colocalization colour mapping

## A tool for analyzing the correlation of the colococalization between two fluorescent channels


**Regression adjusted colocalization colour mapping (RACC)** is a method to spatially visualize colocalization in fluorescence based micrographs using a colourmap. RACC is designed to meet three objective, firstly to highlight regions within a cell that have strong positive correlation between two fluorescence channels, secondly to highlight colocalized regions that have greater combined fluorescence intensities and lastly to remove outliers in the data while maximizing the utilization of the colourmap’s spectrum. These objectives were met using Deming regression along with a penalization factor (θθ ) to suppress voxels for which the underlying fluorescence intensities are less strongly correlated. Outliers are removed by using automatically calculated thresholds.

After regression has been calcualted the colourmap value can be determined with the following formula:

Ci=⎧⎩⎨⎪⎪⎪⎪0,xpi−xp0xpmax−xp0−d tan(θ),1−d tan(θ),if   xpi≤d(xpmax−xp0)tan(θ)+xp0  or  d>dtif   d(xpmax−xp0)tan(θ)+xp0<xpi<xpmaxif   xpi≥xpmaxCi={0,if   xpi≤d(xpmax−xp0)tan(θ)+xp0  or  d>dtxpi−xp0xpmax−xp0−d tan(θ),if   d(xpmax−xp0)tan(θ)+xp0<xpi<xpmax1−d tan(θ),if   xpi≥xpmax

For a full derivation and explanation please refer to the paper (submitted, acceptance pending).

## Download and installation

The RACC utility can be downloaded from here (requires Python 3.5+):

- RACC v0.8 ZIP file (includes sample files)

### Quick installation (Windows only)

Simply run the included .bat files to install all the packages as well as run RACC.

### Manual installation (Windows, Linux, MacOS)

It currently has the following dependencies, that can be installed by running the following `pip` commands in the terminal/command prompt.

```
pip install numpy
pip install matplotlib
pip install scikit-image
pip install scipy
pip install mayavi
pip install PyQt5
```

You can then run RACC by typing the following into terminal/command prompt, while you are in the same path as where `RACC.py` is stored.

```
python RACC.py
```

## Using this RACC utility

### Input file types

RACC takes **two single channel** fluorescence images or stacks as input - image can still be RGB, but should not contain more than one fluorescent label. The input type of 2D images or single slices can be any of the common file types (PNG, JPG, TIFF, etc.). 3D image stacks should be provided in TIFF format (separate slices can be combined into a TIFF file using software like ImageJ/Fiji). *Note:* In the fluorescence channel visualization in 3D the first provided stack will be visualized in Red and the second stack will be visualized in Green, this does not affect the analysis.

### Utility settings

In the RACC utility you select the two input files (both must be either 2D or 3D). You can optionally view the input images which will be displayed as soon as you click “Process”.

Under the parameters group, you can set the background filtering thresholds for the individual channels. The penelization factor, which diminishes voxels that are far from the regression line. The precentage to include is used to automatically calculate both a distance threshold away from the regression line, and a maximum combined intensity (all voxels beyond these thresholds are excluded from the resulting visualization).

In the output settings, you can select the file type, whether it should use the built in colourmap or output the image as grayscale. You can also optionally visualize the output within the utility.

After all the settings are as desired, click on “Process” to calculate the RACC output for the given input.

## Report errors

Please note that this utility is not in active development, however, please report any problems with the utility to rensutheart22 (at) gmail.com
