# Supplementary figures and images for: Regression adjusted colocalisation colour mapping (RACC): A novel biological visual analysis method for qualitative colocalisation analysis of 3D fluorescence micrographs
Source: PLoS One. 2019 Nov 11;14(11):e0225141. doi: 10.1371/journal.pone.0225141 (PMC6844467; doi:10.1371/journal.pone.0225141)

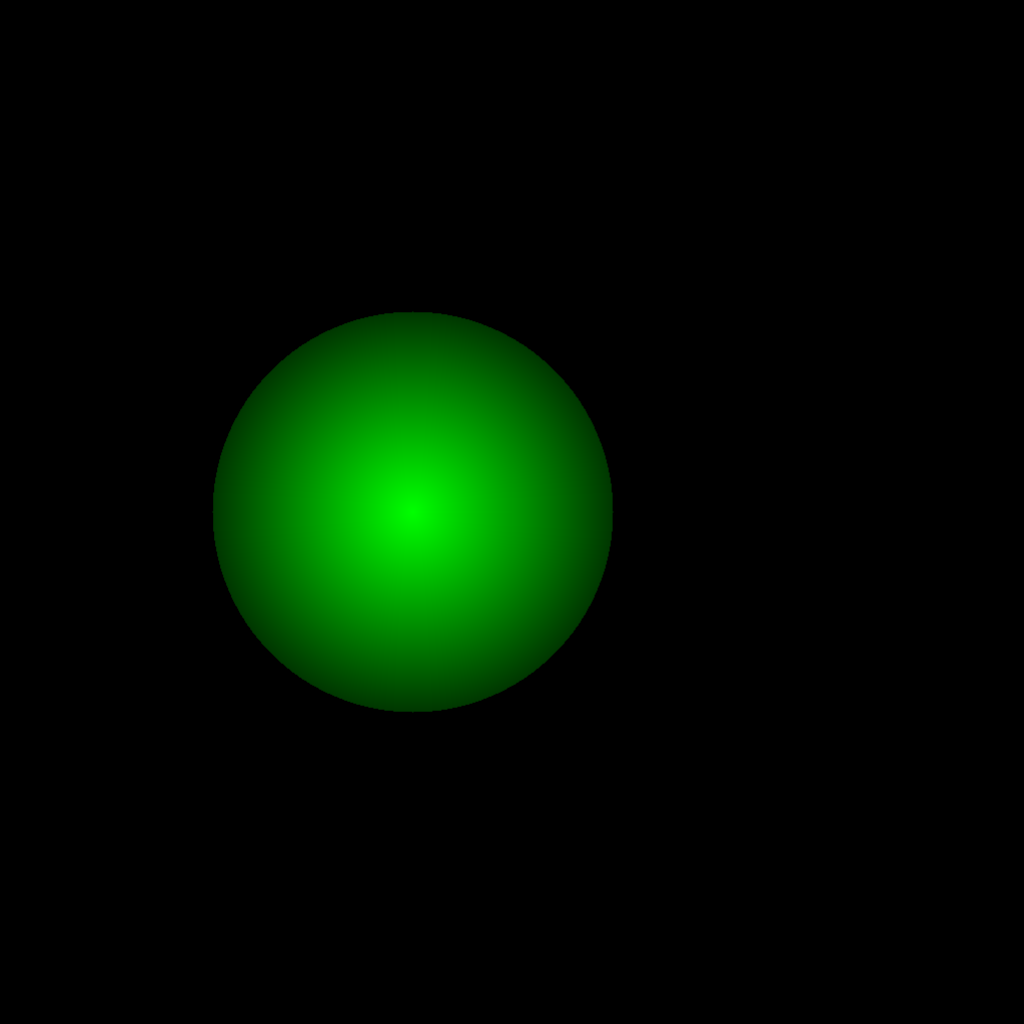

Supplement: S1 File — (ZIP) [file pone.0225141.s002.zip › RACC_v0.8/Green2D.png]

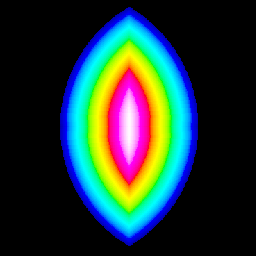

Supplement: S1 File — (ZIP) [file pone.0225141.s002.zip › RACC_v0.8/icon.png]

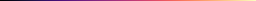

Supplement: S1 File — (ZIP) [file pone.0225141.s002.zip › RACC_v0.8/magmaLine.png]

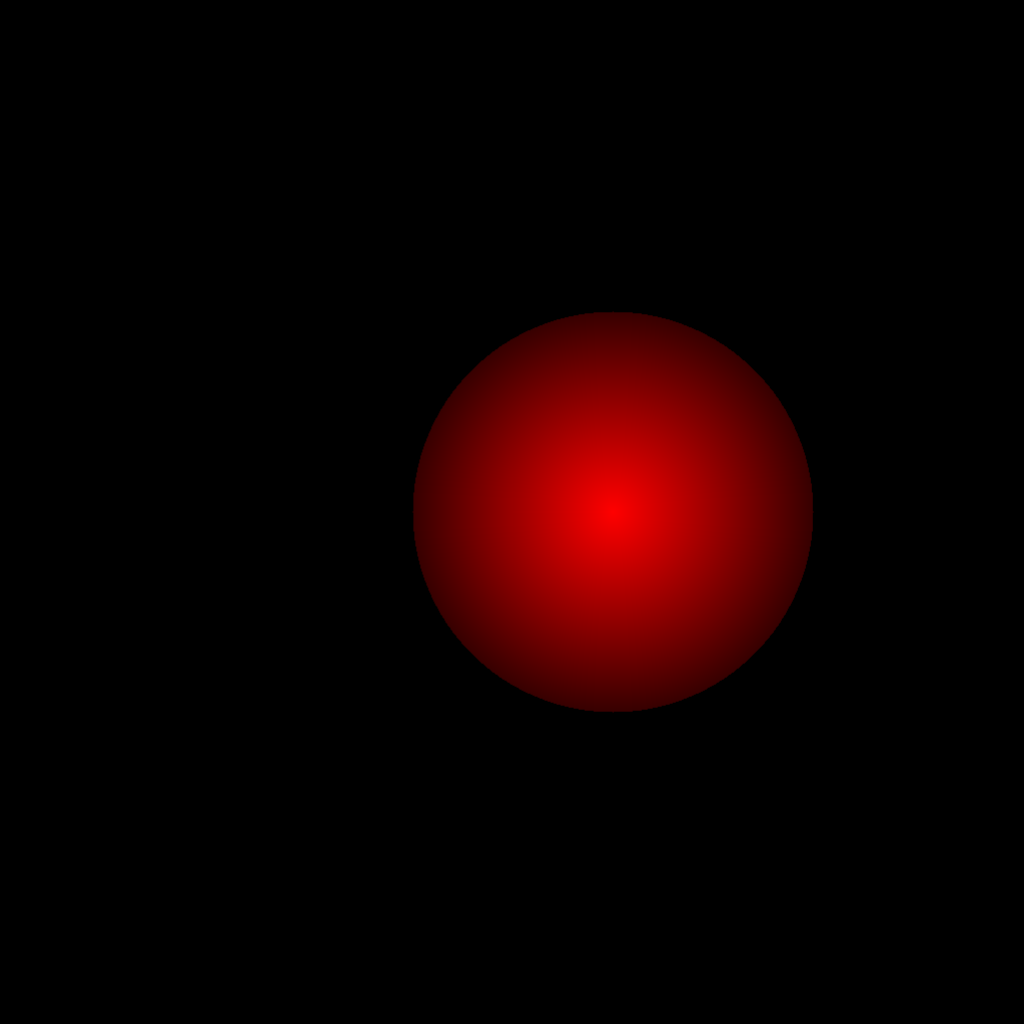

Supplement: S1 File — (ZIP) [file pone.0225141.s002.zip › RACC_v0.8/Red2D.png]
